# To the Medical Profession

**Published:** 1882-04

**Authors:** 


### ARTICLE XXXII.

### TO THE MEDICAL PROFESSION.

"The *Pharmacist* desires to know what is your code of ethics, —to what are you willing to subscribe? We were formerly somewhat certain of what your code of ethics was, but recently we have had so many of the old pillars of the "æsthetic" structure rudely demolished that we have to ask for information as to the portion that does remain. Does your code of ethics forbid you to prescribe a patent medicine—a medicine the working formula of which is unknown, the ingredients of which in their *exact* proportion is not known? A medicine, the name of which can be used only by one manufacturer?

"Is it in accord with your code of ethics to prescribe Listerine, Safoline, Purifinis, Bromo-Chloralum, Lactopeptine, Firwein, Diptherine, Dextro-Quinine, Bromidia, Iodia?

"Is it necessary for you, in order to conform to the code of ethics, to know what is in the medicine you prescribe? If so what are the ingredients of any or all of the ten articles mentioned above? Could you and a practical pharmacist produce the same by applying all your knowledge of it together with his pharmaceutical knowledge?

"If not necessary to know what you are prescribing, is it contrary to the spirit of the code of ethics to prescribe Jayne's Expectorant, Bull's Sarsaparilla, Helmbold's Buchu, Injection Brou, Tarrant's Extract of Cubebs and Copabia, Warner's Kidney and Liver Cure? If this is not contrary to your code of ethics, why do you object to the druggist selling them?

"The *Pharmacist* truly states that it does not know on what code of ethics the medical profession stands, and is all at sea when it attempts to set a bound beyond which the medical profession will not go.

"Gentlemen, is this not true? and is it not your duty to help your brother profession to the extent of defining your position?'
—*Pharmacist and Chemist.*

In reply to the above numerous questions asked by the *Pharmacist and Chemist*, of this City, we answer, emphatically, that both the Code of Ethics and the *Code of Common Sense*, *do forbid* the prescribing of any " patent medicine,"—or medicine " the working formula for which is unknown,"—or, " the name of which can be used only by one manufacturer."

In other words our professional Code of Ethics is strictly opposed, both in letter and spirit, to all use of patents, trade marks, copy-rights, or secret processes in the manufacture and sale of medicines to be used for the treatment of disease. We do not knowingly use medicines thus restricted, and for years have refused even to look at them when thrust before us by the numerous agents traveling over the country with their *elegant* samples.

There is but one plain, honest, scientific rule for the guidance of both physician and pharmacist; namely, the former while at the bed-side of his patient, should determine with careful discrimination, not only the exact composition but also the relative proportion of each constituent of the prescription he makes, that both may be accurately adjusted to the wants of the individual patient, while the latter should exercise all his skill in the direction of furnishing pure medicines of standard strength, and in forms most reliable for preservation and convenient for use. The whole modern flood of proprietary medical compounds, is, to the community, a most expensive luxury, and to the medical profession a positive nuisance.

---

MASTURBATION.—Dr. William A. Hammond, in a letter to the *Louisville Medical News*, says that masturbation is "a vile, vicious, degrading and demoralizing practice, that no one could be guilty of without self-abasement," but that so far as the generative organs are concerned; so far as the physical effects go, it is "no more injurious than a corresponding amount of sexual intercourse. Young men and boys injure themselves by the act, because the facilities for performing it are greater than they are for indulging in sexual intercourse." Prof. Hammond is an authority on this subject, and his assertions will set at rest many disquieted minds.

